# Implications of construction method and spatial scale on measures of the built environment

**DOI:** 10.1186/s12942-016-0044-x

**Published:** 2016-04-28

**Authors:** Julie Strominger, Rebecca Anthopolos, Marie Lynn Miranda

**Affiliations:** School of Natural Resources and the Environment, University of Michigan, Ann Arbor, MI 48109 USA; Children’s Environmental Health Initiative, Rice University, Houston, TX 77005 USA; Department of Statistics, Rice University, 6100 Main Street, MS-2, Houston, TX 77005 USA; Department of Pediatrics, University of Michigan, Ann Arbor, MI 48109 USA; Department of Pediatrics, Baylor College of Medicine, Houston, TX 77030 USA; Department of Pediatrics, Duke University, Durham, NC 27708 USA

**Keywords:** Built environment, Neighborhood measures, Construction method, Spatial scale

## Abstract

**Background:**

Research surrounding the built environment (BE) and health has resulted in inconsistent findings. Experts have identified the need to examine methodological choices, such as development and testing of BE indices at varying spatial scales. We sought to examine the impact of construction method and spatial scale on seven measures of the BE using data collected at two time points.

**Methods:**

The Children’s Environmental Health Initiative conducted parcel-level assessments of 57 BE variables in Durham, NC (parcel N = 30,319). Based on a priori defined variable groupings, we constructed seven mutually exclusive BE domains (housing damage, property disorder, territoriality, vacancy, public nuisances, crime, and tenancy). Domain-based indices were developed according to four different index construction methods that differentially account for number of parcels and parcel area. Indices were constructed at the census block level and two alternative spatial scales that better depict the larger neighborhood context experienced by local residents: the primary adjacency community and secondary adjacency community. Spearman’s rank correlation was used to assess if indices and relationships among indices were preserved across methods.

**Results:**

Territoriality, public nuisances, and tenancy were weakly to moderately preserved across methods at the block level while all other indices were well preserved. Except for the relationships between public nuisances and crime or tenancy, and crime and housing damage or territoriality, relationships among indices were poorly preserved across methods. The number of indices affected by construction method increased as spatial scale increased, while the impact of construction method on relationships among indices varied according to spatial scale.

**Conclusions:**

We found that the impact of construction method on BE measures was index and spatial scale specific. Operationalizing and developing BE measures using alternative methods at varying spatial scales before connecting to health outcomes allows researchers to better understand how methodological decisions may affect associations between health outcomes and BE measures. To ensure that associations between the BE and health outcomes are not artifacts of methodological decisions, researchers would be well-advised to conduct sensitivity analysis using different construction methods. This approach may lead to more robust results regarding the BE and health outcomes.

**Electronic supplementary material:**

The online version of this article (doi:10.1186/s12942-016-0044-x) contains supplementary material, which is available to authorized users.

## Background

The built environment (BE) is defined as the “human-made space in which people live, work, and recreate on a day-to-day basis” and includes the physical condition of homes, outdoor spaces, roads, sidewalks, and schools [1]. Previous research has found poor quality BE, measured by domains such as housing quality and nuisances, to be adversely associated with a multitude of human health outcomes, such as preterm birth [2], mental health [3–7], and childhood weight status [8, 9]. Despite evidence of a deleterious relationship between BE and health, findings remain inconsistent, with several studies showing null associations [10–13]. Such results have led researchers to hypothesize that inconsistent findings may be an artifact of methodological choices, thus prompting a call for an examination of methodology in the development of measures of the BE [12, 14–16].

Existing studies have documented the varying ways of first measuring and then operationalizing BE measurement in health outcomes research. In constructing BE measures of the physical neighborhood environment, source data have been derived from perceived and observed data [17], with subsequent metrics developed using geographic information system (GIS) methods and data reduction techniques [12, 14, 17]. Additionally, spatial scale has been identified as a source of variation among BE measures [12, 15], with BE measures largely operationalized based on administratively-defined geographic units like census tracts or tax parcels [18]. Alternatively, researchers may construct neighborhoods based on community-defined neighborhood boundaries [19]. Thus, in health outcomes research, the potential for methodological choice to substantively impact study findings is widely understood [12, 14, 20, 21], and in light of methodological heterogeneity, the difficulty in making inter-study comparisons is not surprising [15].

To date, while researchers frequently examine the impact of certain methodological choices in constructing BE measures on a given association of scientific interest, for example, by estimating associations at alternative buffer sizes or census geographic units [18, 22, 23], to our knowledge, few studies have focused on the consequences of these choices on BE measurement itself [22, 24–26]. The few studies that have investigated the impact on BE measurement are focused on measuring the food environment and green space [25, 27]. Absent from the literature is a systematic assessment of methodological choices in the construction of BE measures related to different BE domains. In this study, we respond to researchers’ calls for a methodological assessment of BE measures in terms of standardization related to underlying geography and spatial scale of measurement [12, 15, 16]. Using objective survey data from a BE assessment tool conducted in Durham, NC during 2008 and again in 2011, combined with supplemental administrative data on renter occupancy tenure and crime, we develop seven BE indices (housing damage, property disorder, territoriality, vacancy, public nuisances, crime, and tenancy) according to four different index construction methods that alternatively account for number of parcels and parcel area. We apply three spatial scales that differentially account for the spatial structure of the study area. We investigate the implications of construction method and spatial scale on BE measures.

## Methods

### Study area

**Fig. 1:**
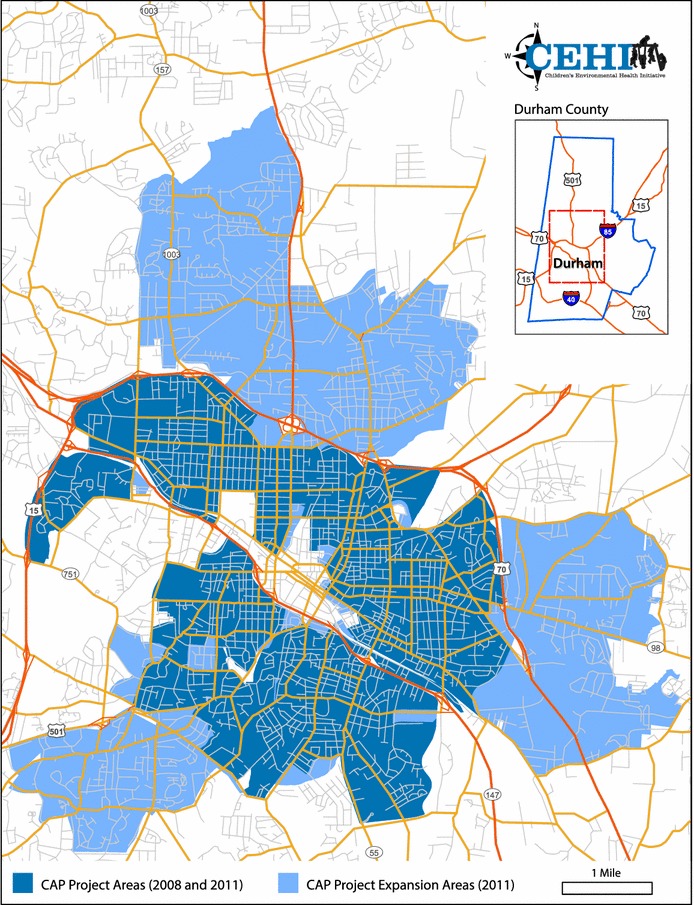
Map of the Community Assessment Project (CAP) study area, 2008 and 2011

Figure 1 presents the study area that encompasses the urban core of Durham, North Carolina. In 2008, the study area comprised 886 census blocks (N = 17,225 parcels); in 2011, the study area was enlarged to include additional contiguous census blocks (total N = 1380 census blocks, 31,839 parcels).

### Objective tax-parcel survey data

The design and data collection for the Community Assessment Project (CAP) has been described in detail elsewhere [28]. An objective tax-parcel level survey, the CAP was built using a GIS data systems architecture. Equipped with handheld global positioning systems units, teams of trained raters collected data from the sidewalk or street. Fifty-seven variables, determined based on literature review and feedback from the Durham community, were recorded [29–31]. Variables related to land use; occupancy status; the presence of nuisances such as litter and graffiti; evidence of territoriality such as barbed wire and fencing; and the physical condition of any buildings, yard, or property, were documented. Residential, commercial, and other property types were similarly assessed. Excluding land use and occupancy status, variables were assessed for presence or absence for each parcel in the study area. Land use was recorded as commercial, community, empty lot, faith, government, parking lot, property, or residential type. For occupancy status, each parcel was recorded as either unoccupied or occupied.

The inter-rater reliability (IRR) of the CAP data was calculated across seven raters for each of the variables using 2011 data. The average agreement over the variables was 0.95 (95 % CI 0.945, 0.953), well-above the conventional threshold of 0.70 for strong agreement [32]. While IRR was not computed for 2008, the same supervisor administered the training, and materials and modules were consistent between time periods, which would suggest a similarly strong IRR in 2008.

### Supplementary data

To correspond to the CAP in 2008 (2011), we used 2008 (2011) crime and 2008 (2010) tax assessor data from the Durham Police Department and Durham Tax Assessor’s office, respectively. Crime data were geocoded to the census block level and aggregated to yield counts of total crime per block. Tenure status was derived from the tax assessor data for each residential parcel by comparing the owner’s address in the record to the geographic address. Parcels were marked as owner-occupied when the physical and owner addresses matched and renter-occupied otherwise. The matching algorithm accounted for discrepancies such as typos and spelling errors.

### Variable groupings

We grouped the CAP survey variables into five distinct domains: housing damage (e.g., boarded doors and roof damage), property disorder (e.g., litter and broken glass), territoriality (e.g., fencing and security signs), vacancy, and public nuisances (e.g., graffiti and cigarette butts) (Additional file 1: Table S1). Nuisances that were on or within 2 feet of public space were recorded as public nuisances, while nuisances that were on private property beyond 2 feet from public space were recorded as property disorder.

### Spatial scale

**Fig. 2:**
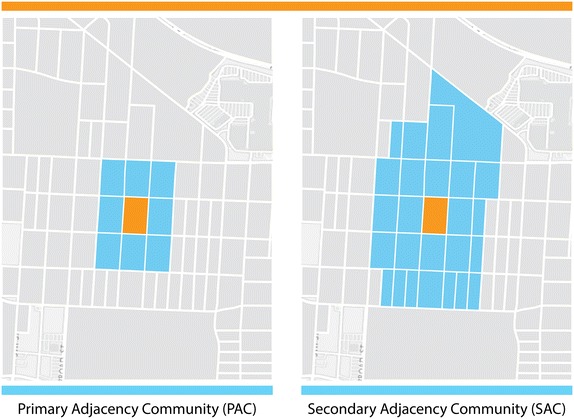
Primary and secondary adjacency communities. This figure depicts the primary adjacency community (PAC) in the *left* frame and secondary adjacency community (SAC) in the *right* frame

We constructed the seven BE indices at the census block level and two alternative spatial scales defined by adjacency. The first, primary adjacency community (PAC), refers to the index block along with adjacent blocks that share a boundary, in the form of a vertex or line segment. The PAC is specified by constructing a first order adjacency matrix. Explicitly, let *W* be a symmetric matrix with dimensions equal to the total number of blocks in the study area. If census blocks *j* and *i* share a vertex or line segment, then entry *w*_*ij*_ = 1; otherwise, *w*_*ij*_ = 0. Since in our analytical context, we consider a block to be a neighbor to itself, we set the diagonal entries *w*_*ii*_ = 1. The secondary adjacency community (SAC) extends the PAC by additionally including secondary neighbors (see Fig. 2) in the adjacency matrix construction. The mean geographic area among census blocks was 0.05 square kilometers (SD = 0.10). For PAC and SAC, the mean geographic area was 0.52 (SD = 0.55) and 1.56 (SD = 13.39) square kilometers, respectively.

### Alternative index constructions

The four index construction methods, hereafter referred to as Methods 1–4, are based on alternative approaches to standardizing the variables (e.g., litter of the public nuisances index) composing each BE index. Methods 1–4 are replicated for each of the three spatial scales. For a given method and spatial scale, standardized variables representing each domain are summed together, yielding a BE domain-specific score (e.g., standardized litter + standardized graffiti + standardized garbage, etc. = property disorder measure). Below, we explicate the development of Methods 1–4 using Method 1 as the heuristic example.

Method 1 aggregates each variable to a given spatial scale, resulting in a count (e.g., count of parcels with litter present in a census block). This count is then standardized to have mean 0 and standard deviation (SD) 1. Let *c*_*bp*_ be an indicator for the presence of variable *c* in the *p*th parcel in spatial unit *b*. Then, for the $p = 1, \ldots ,P_{b}$ parcels in spatial unit *b*, the count of a given variable present for all parcels in the index spatial unit is defined as

1 $$c_{b} = \mathop \sum \limits_{p = 1}^{{P_{b} }} c_{bp} .$$

We then standardize this count by subtracting the average count over the spatial units in the study region and dividing by the SD in the study region. Explicitly, the standardized count for a given variable is

2 $$c_{b}^{Std} = \frac{{c_{b} - \bar{c}}}{{\sqrt {var(c)} }},$$

such that $$c_{b}^{Std} \sim N(0,1).$$

Within each BE domain, the standardized counts of variables are summed to yield an index value for each spatial unit. Formally, for a given BE domain *BE* in spatial unit *b*, we have

3 $$BE_{b} = \mathop \sum \limits_{j = 1}^{J} c_{bj}^{Std} \quad {\text{for}}\;\; j = 1, \ldots ,J\,{\text{variables}} .$$

Method 2 extends Method 1 by dividing the count by the number of parcels in a given spatial unit, resulting in an average count per parcel (e.g., average count of litter per parcel in a census block). Instead of number of parcels, Method 3 analogously accounts for geographic area, resulting in an average count per unit area (e.g., average count of litter per unit area in a census block). Method 4 uses an alternative approach to account for the underlying area by aggregating the area with a variable present over the spatial unit and then dividing by the total area, resulting in the proportion of area in a spatial unit with a variable present (e.g., the proportion of the total area in a census block with litter present). As in Method 1, variables composing a given BE measure are summed to yield a BE domain-specific score. Table 1 summarizes the different construction methods.

**Table 1:** Definitions of alternative construction methods for one variable

Method	Definition
Method 1	Count of parcels with variable present
Method 2	$\frac{{{\text{Count }}\,{\text{of}}\,{\text{parcels}}\,{\text{with}}\,{\text{variable }}\,{\text{present}} }}{{{\text{Total}}\,{\text{number}}\,{\text{of}}\,{\text{parcels}}}}$
Method 3	$\frac{{{\text{Count }}\,{\text{of}}\,{\text{parcels}}\,{\text{with}}\,{\text{variable }}\,{\text{present}} }}{{{\text{Total}}\,{\text{area}}}}$
Method 4	$\frac{{{\text{Total}}\,{\text{area}}\,{\text{with}}\,{\text{variable }}\,{\text{present}} }}{{{\text{Total}}\,{\text{area}}}}$

For example, security bars is one variable that contributes to the territoriality index. Additional file 1: Table S1 details which variables contribute to each index

### Statistical analyses

For all BE indices except public nuisances and tenancy, tax parcels that could not be fully assessed due to factors like view obstruction were removed (remaining N for 2008 = 16,608; N for 2011 = 30,319). Further, 15,582 and 28,320 parcels were assessed for public nuisances in 2008 and 2011, and 16,040 and 29,256 parcels were assessed for tenancy in 2008 and 2011, respectively. Indices were constructed based on census block boundaries from the 2010 US Census. All BE indices were based on tax parcel area with the exception of public nuisances and crime. Since public nuisances were defined by their location on or within 2 feet of public property, parcel frontage area was used. The crime index was constructed based on census block area, as these data were available only at the census block level. All BE indices were constructed at the three spatial scales using Methods 1–4 except for crime, which was calculated using only Methods 1 and 3 since parcel-level data were unavailable.

To evaluate if block rank was preserved across Methods 1–4, we computed Spearman’s rank correlation among alternatively-constructed measures of the same BE index using block-level measures (e.g., housing damage indices constructed according to Methods 1–4). In order to assess how well block rankings were preserved, the mean rank among alternatively-constructed measures of the same index was computed for each census block. We then calculated the average absolute difference from the mean rank for each block, resulting in an index-specific average mean absolute difference (MAD) in rank. The MAD for each index was then mapped to identify blocks where rank was sensitive to construction method. Further, we calculated the average MAD for each index to enable inter-index comparisons of preservation. We analogously used Spearman’s rank correlation to investigate whether associations among indices were preserved across Methods 1–4. For example, we compared the association between housing damage and property disorder based on Method 1 with that based on Method 4. To investigate the implications of spatial scale on BE indices, we replicated our analysis at the PAC and SAC levels.

ArcGIS version 10.2 (ESRI, Redlands, CA, USA) was used to compute block, parcel, and parcel frontage area. The *rgdal* package in R 3.0.1 (The R Foundation for Statistical Computing, 2013) was used to import the ArcGIS shapefile into R and the *spdep* package was used to create the adjacency matrices. R code that creates adjacency matrices for the PACs and SACs from a shapefile can be found in Additional file 2. SAS 9.4 was used to clean the data, create the indices, and conduct the statistical analysis (SAS Institute, Cary, NC, USA).

## Results

Analysis presented in the main text is based on census block level calculations using 2011 data. Tables related to the PAC and SAC levels, along with 2008 results, can be found in Additional file 1.

The mean, SD, minimum, and maximum of the seven BE indices for each construction method are presented in Table 2. Among the four BE indices made up of more than one variable (housing damage, property disorder, territoriality, and public nuisances), Method 1, which was based on a simple count, resulted in the largest variation. Variation in indices constructed using Method 3, which accounted for parcel area, was greater than variation in indices constructed using Method 2, which accounted for number of parcels, with the exception of housing damage. Variation based on Method 4 was index specific. Crime measures constructed from a simple count (Method 1) resulted in a larger range than those standardized by area (Method 3), while the range of vacancy and tenancy was greatest when constructed using Methods 1 or 3.

**Table 2:** Summary statistics of the seven built environment indices by method, census block level, 2011 (N = 1380)

	Mean (SD)	Minimum–maximum
Method 1		
Housing damage	0 (6.55)	−3.05 to 88.71
Property disorder	0 (8.47)	−5.68 to 102.36
Territoriality	0 (4.05)	−3.24 to 41.21
Vacancy	0 (1.00)	−0.54 to 12.00
Public nuisances^a^	0.04 (11.51)	−9.2 to 127.75
Crime	0 (1.00)	−0.38 to 24.89
Tenancy^b^	0 (1.00)	−0.75 to 15.42
Method 2		
Housing damage	0 (5.80)	−2.89 to 106.24
Property disorder	0 (6.22)	−5.90 to 65.87
Territoriality	0 (2.84)	−4.27 to 34.81
Vacancy	0 (1.00)	−0.61 to 4.76
Public nuisances^a^	−0.05 (8.10)	−7.95 to 80.85
Crime^c^	–	–
Tenancy^b^	0 (1.00)	−1.89 to 1.51
Method 3		
Housing damage	0 (5.75)	−2.79 to 56.91
Property disorder	0 (6.83)	−5.21 to 55.50
Territoriality	0 (3.32)	−3.52 to 34.24
Vacancy	0 (1.00)	−0.46 to 12.34
Public nuisances^a^	0.04 (8.9)	−9.64 to 64.00
Crime	0 (1.00)	−0.74 to 9.48
Tenancy^b^	0 (1.00)	−0.88 to 15.86
Method 4		
Housing damage	0 (5.32)	−2.61 to 79.35
Property disorder	0 (5.90)	−5.4 to 56.71
Territoriality	0 (2.90)	−3.99 to 30.96
Vacancy	0 (1.00)	−0.6 to 4.55
Public nuisances^a^	−0.04 (8.36)	−8.57 to 68.10
Crime^c^	–	–
Tenancy^b^	0 (1.00)	−1.89 to 1.40

Method 1 is a simple count, Method 2 is an average count per parcel, Method 3 is an average count per unit area, and Method 4 is proportion of area with a variable present

*SD* standard deviation

^a^N for public nuisances is 1356 due to data availability

^b^N for tenancy is 1358 due to data availability

^c^Crime is not constructed using Methods 2 or 4 as crime is measured at the block level

Correlations among alternatively-constructed measures of the same index are presented in Table 3. Consistent with previous research [33–35], we used the following categories to evaluate rank preservation: a correlation ≥0.7 indicated a well preserved index, a correlation ≥0.5 and <0.7 indicated a moderately preserved index, a correlation ≥0.3 to <0.5 indicated a weakly preserved index, and a correlation <0.3 indicated a very weakly or not preserved index. We observe that housing damage, property disorder, vacancy, and crime were well preserved across Methods 1–4 (*ρ* = 0.91–0.98, 0.77–0.94, 0.89–0.96, and 0.78, respectively), while territoriality and public nuisances were moderately to well preserved across methods (*ρ* = 0.60–0.90 and 0.54–0.94, respectively). Preservation of tenancy depended on a given pairwise comparison between methods (*ρ* = 0.25–0.94). Comparing tenancy constructed using Methods 2 and 4 suggested a well preserved index (*ρ* = 0.94), while comparing Methods 1 and 2 or 4 indicated a very weakly preserved index (*ρ* = 0.25–0.26, respectively). All other pairwise comparisons suggested a weakly preserved index (*ρ* = 0.36–0.46).

**Table 3:** Spearman’s correlations between alternatively-constructed measures of each index, census block level, 2011 (N = 1380)

	(1)	(2)	(3)	(4)
Housing damage				
Method 1 (1)	1.00	0.93	0.92	0.91
Method 2 (2)		1.00	0.97	0.98
Method 3 (3)			1.00	0.96
Method 4 (4)				1.00
Property disorder				
Method 1	1.00	0.80	0.79	0.77
Method 2		1.00	0.88	0.94
Method 3			1.00	0.85
Method 4				1.00
Territoriality				
Method 1	1.00	0.66	0.61	0.60
Method 2		1.00	0.73	0.90
Method 3			1.00	0.69
Method 4				1.00
Vacancy				
Method 1	1.00	0.91	0.89	0.91
Method 2		1.00	0.94	0.96
Method 3			1.00	0.92
Method 4				1.00
Public nuisances^a^				
Method 1	1.00	0.54	0.80	0.56
Method 2		1.00	0.71	0.94
Method 3			1.00	0.67
Method 4				1.00
Crime^b^				
Method 1	1.00	–	0.78	–
Method 3			1.00	–
Tenancy^c^				
Method 1	1.00	0.25	0.46	0.26
Method 2		1.00	0.46	0.94
Method 3			1.00	0.36
Method 4				1.00

Method 1 is a simple count, Method 2 is an average count per parcel, Method 3 is an average count per unit area, and Method 4 is proportion of area with a variable present

^a^N for public nuisances is 1356 due to data availability

^b^Crime is not constructed using Methods 2 or 4 as crime is measured at the block level

^c^N for tenancy is 1358 due to data availability

**Fig. 3:**
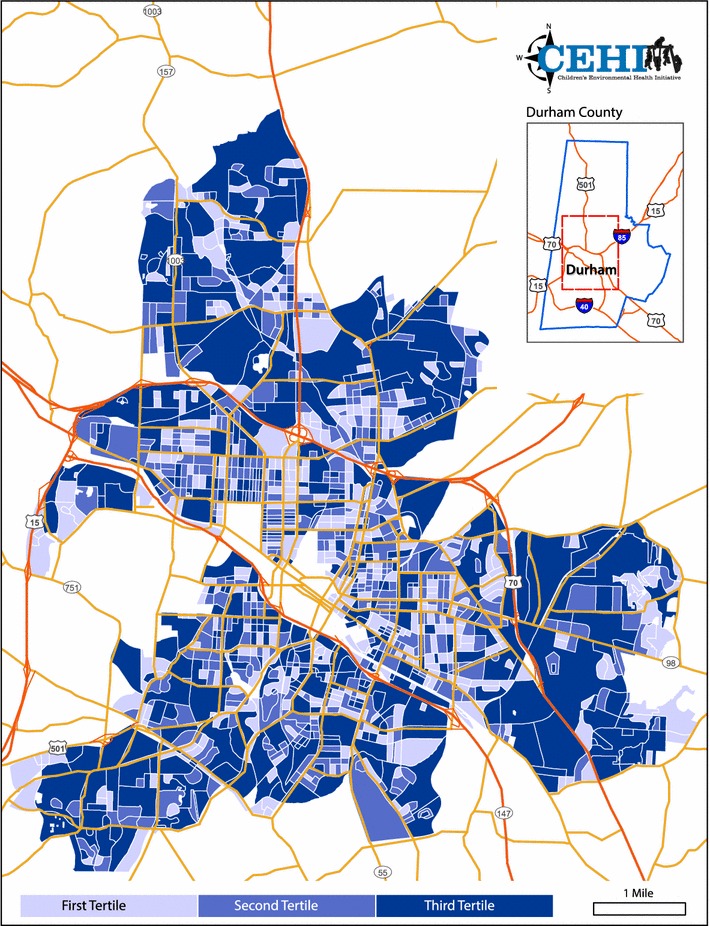
Spatial distribution of the mean absolute difference (MAD) in census block rank for territoriality, 2011. The *first tertile* represents blocks weakly impacted by method, the *second tertile* represents blocks moderately impacted by method, and the *third tertile* represents blocks strongly impacted by method

The mean and SD of the average MAD for each BE index are presented in Table 4. Larger means indicate that block rank was more heavily impacted by construction method. Interpreted as the average difference from the mean block rank, the highest average MAD values were for tenancy (mean = 187.03, SD = 133.02), public nuisances (mean = 134.38, SD = 103.88), and territoriality (mean = 134.12, SD = 100.74). The lowest average MAD values were for housing damage (mean = 45.45, SD = 54.31) and vacancy (mean = 52.98, SD = 64.28). It is important to note that the high MAD values correspond to a roughly 14 % difference in ranks; whereas the lowest MAD value corresponds to a roughly 3 % difference in ranks. Figure 3 presents an example of the spatial distribution of MAD using territoriality.

**Table 4:** Summary statistics of index-specific mean absolute difference (MAD) in rank, census block level, 2011 (N = 1380)

Index	Mean (SD)
Housing damage	45.45 (54.31)
Property disorder	94.44 (76.92)
Territoriality	134.12 (100.74)
Vacancy	52.98 (64.28)
Public nuisances^a^	134.38 (103.88)
Crime	89.48 (96.59)
Tenancy^b^	187.03 (133.02)

*SD* standard deviation

^a^N for public nuisances is 1356 due to data availability

^b^N for tenancy is 1358 due to data availability

Table 5 presents the correlations among indices for each construction method. Preservation of relationships among different indices (e.g., housing damage and property disorder) implies similar correlations independent of construction method. Since we are not aware of any a priori defined thresholds for assessing the preservation of a relationship between BE indices across methods, for consistency, we use the same categories as previously mentioned. If the strength of the relationship between two indices varies across Methods 1–4 (e.g., the relationship between housing damage and property disorder is weak using Method 1 and moderate using Method 2), the relationship is considered to be weakly preserved. If the strength of the relationship is consistent across Methods 1–4, it is considered to be well preserved. Correlations between crime and housing damage, territoriality, or public nuisances, and public nuisances and tenancy were indicative of well preserved relationships across Methods 1–4, while relationships among all other indices were weakly preserved. For example, the relationship between property disorder and territoriality was very weak using Methods 2 or 4 (*ρ* = 0.28 and 0.25, respectively) and moderate using Methods 1 or 3 (*ρ* = 0.68 and 0.61, respectively). In general, relationships appeared to be well preserved when comparing indices constructed using Method 2 that accounted for number of parcels to Method 4 that accounted for proportion of the area with a variable present, or Method 1 based on a simple count to Method 3 that accounted for area.

**Table 5:** Spearman’s correlations among indices by method, census block level, 2011 (N = 1380)

	Housing damage	Property disorder	Territoriality	Vacancy	Public nuisances	Crime	Tenancy
Method 1							
Housing damage	1.00	0.72	0.55	0.47	0.59	0.42	0.55
Property disorder		1.00	0.68	0.57	0.69	0.53	0.68
Territoriality			1.00	0.43	0.58	0.48	0.62
Vacancy				1.00	0.55	0.34	0.61
Public nuisances					1.00	0.46	0.66
Crime						1.00	0.54
Tenancy							1.00
Method 2							
Housing damage	1.00	0.58	0.26	0.29	0.29	–	0.20
Property disorder		1.00	0.28	0.35	0.42	–	0.32
Territoriality			1.00	−0.03	0.05	–	−0.22
Vacancy				1.00	0.27	–	0.31
Public nuisances					1.00	–	0.55
Crime^a^						–	–
Tenancy							1.00
Method 3							
Housing damage	1.00	0.72	0.55	0.41	0.56	0.44	0.60
Property disorder		1.00	0.61	0.47	0.63	0.45	0.68
Territoriality			1.00	0.20	0.43	0.35	0.49
Vacancy				1.00	0.44	0.19	0.48
Public nuisances					1.00	0.39	0.59
Crime						1.00	0.40
Tenancy^a^							1.00
Method 4							
Housing damage	1.00	0.56	0.25	0.27	0.26	–	0.12
Property disorder		1.00	0.25	0.30	0.39	–	0.24
Territoriality			1.00	−0.04	0.06	–	−0.24
Vacancy				1.00	0.24	–	0.26
Public nuisances					1.00	–	0.50
Crime^a^						–	–
Tenancy							1.00

Method 1 is a simple count, Method 2 is an average count per parcel, Method 3 is an average count per unit area, and Method 4 is proportion of area with a variable present

N for the pairwise comparison between public nuisances and tenancy is 1336, N for other pairwise comparisons involving public nuisances is 1356, and N for other pairwise comparisons involving tenancy is 1358 due to data availability

^a^Crime is not constructed using Methods 2 or 4 as crime is measured at the block level

To evaluate whether our findings were consistent across spatial scale, we replicated our analysis at the PAC and SAC levels. Findings were reasonably consistent across spatial scale when assessing index-specific preservation across methods for housing damage, territoriality, public nuisances, and tenancy (Additional file 1: Tables S2, S3). Crime and vacancy, which were well preserved across Methods 1–4 at the block level (*ρ* = 0.78 and 0.89–0.96, respectively), were less preserved at the PAC (*ρ* = 0.45 and 0.58–0.84, respectively) and SAC levels (*ρ* = 0.29 and 0.54–0.88, respectively). Property disorder, which was well preserved across Methods 1–4 at the block and PAC levels (*ρ* = 0.77–0.94 and 0.72–0.90, respectively), was moderately to well preserved at the SAC level (*ρ* = 0.68–0.93).

Relationships between indices that were preserved across Methods 1–4 at the block level were weakly preserved across methods at the PAC and SAC levels, while a majority of the relationships that were impacted at the block level remained impacted at the PAC and SAC levels (Additional file 1: Tables S5, S6). We observe that the relationships between housing damage and crime, and public nuisances and tenancy, which were well preserved across methods at the block level (*ρ* = 0.42–0.44 and 0.50–0.66, respectively), were weakly preserved across methods at the PAC (*ρ* = 0.40–0.63 and 0.37–0.77, respectively) and SAC levels (*ρ* = 0.38–0.70 and 0.57–0.87, respectively). Further, the relationships between crime and territoriality or public nuisances were well preserved across methods at the block (*ρ* = 0.35–0.48 and 0.39–0.46, respectively) and PAC levels (*ρ* = 0.54–0.64 and 0.60–0.64, respectively) but weakly preserved at the SAC level (*ρ* = 0.64–0.70 and 0.63–0.71, respectively). In contrast, the relationships between housing damage and property disorder, and crime and tenancy were weakly preserved across methods at the block level (*ρ* = 0.58–0.72 and 0.40–0.54, respectively) but well preserved at the PAC (*ρ* = 0.75–0.88 and 0.62–0.65, respectively) and SAC levels (*ρ* = 0.84–0.93 and 0.72–0.73, respectively). Similarly, the relationships between vacancy and crime, and property disorder and territoriality were weakly preserved across methods at the block (*ρ* = 0.19–0.34 and 0.25–0.68, respectively) and PAC levels (*ρ* = 0.49–0.53 and 0.55–0.82, respectively) but well preserved at the SAC level (*ρ* = 0.54–0.58 and 0.71–0.93, respectively).

Correlations among alternatively-constructed measures of the same index were relatively consistent across years, as were correlations among indices (Additional file 1: Tables S8, S10, S11, S12).

## Discussion

Using objective survey data supplemented with administrative data collected at two time points, we assessed the impact of construction method on the reliability of seven BE indices and evaluated whether findings were consistent across spatial scale. Results indicated that the tenancy index was strongly impacted by construction method at the block level while territoriality and public nuisances indices were moderately impacted. Excluding the relationships between crime and housing damage, territoriality, or public nuisances, and tenancy and public nuisances, relationships among BE indices were impacted by construction method. Extending the block to account for nearby areas, the number of indices impacted by construction method increased as spatial scale increased. Findings involving vacancy, crime, and property disorder were modified at the PAC and SAC levels, often becoming less preserved with increasing spatial scale. Relationships among indices were consistently impacted by construction method across spatial scale or became impacted as spatial scale increased with a few exceptions. The relationships between crime and vacancy or tenancy, and property disorder and housing damage or territoriality appeared to be less impacted by construction method as spatial scale increased.

We found that the impact of construction method on measures of the BE was not only index and spatial scale specific but also depended on which methods we were comparing. These findings are in line with previous research that suggested that the strength of the relationship between alternatively-constructed measures of the food environment depended on the approaches being compared [27]. Additionally, research has demonstrated that methodological decisions such as buffer size affect measures of access to green space or public open space, walkability, and other land use characteristics [25, 36, 37]. Such sensitivity is consequential to using BE indices in applied research. For example, when using a data reduction technique like principal components analysis that relies on the correlation structure to summarize information, construction method and spatial scale may lead to a different number of composite indices, either consistent with a priori hypotheses or not. In turn, associations between BE and health outcomes may be affected.

Our research indicates that how well relationships were preserved varied according to index and spatial scale. Territoriality, public nuisances, and tenancy indices were less well preserved than other indices at the block level. These findings can be ascribed to how often variables contributing to an index were observed and variation in area and number of parcels. As variation in the count of a variable (i.e., graffiti), area, and number of parcels across the study area increases, block rank becomes less preserved. Alternatively, as variation in the count of a variable, area, or number of parcels across the study area decreases, block rank becomes well preserved as all counts of a variable are divided by the same area or number of parcels. Fencing and security signs from the territoriality index, food garbage, cigarettes, broken glass, and high weeds from the public nuisances index, and renter-occupied parcels from the tenancy index were observed more often than variables contributing to the property disorder and housing damage indices. Further, we observed variation in area and number of parcels across the study area. Thus, adjusting block-level counts of territoriality, public nuisances, and tenancy variables by the number of parcels or area impacted the index and corresponding block rank more than that of property disorder and housing damage. This can be seen in Table 4, which shows that MAD is highest for territoriality, public nuisances, and tenancy.

Further, we found that index preservation was spatial scale specific. At the block level, only three indices were affected by construction method while two and three additional indices were also affected at the PAC and SAC levels, respectively. Variation in index preservation across spatial scale is likely driven by relative dissimilarity among neighboring units. For example, crime, which appeared reliable at the block level (*ρ* = 0.78), was less reliable at the PAC and SAC levels (*ρ* = 0.45 and 0.29, respectively), indicating that as spatial scale increased, the difference in crime measures due to construction method increased in magnitude. Research suggests that crime is clustered, with areas of high crime in close proximity to areas of low crime [38]. Consequently, accounting for crime and area in adjacent blocks, as PAC and SAC level measures do, can significantly impact the rank of a given block, with the magnitude depending on how dissimilar nearby blocks are. Further, heterogeneity among neighboring units can explain why certain relationships among indices were affected differentially across spatial scale. Accounting for adjacent blocks affected index-specific block rank which, in turn, affected relationships among indices.

Results suggested that certain BE indices and relationships among BE indices were better preserved when comparing Methods 1 and 3, and Methods 2 and 4, albeit to a lesser extent when comparing Methods 1 and 3. Measures constructed using Method 1 are equivalent to those constructed using Method 3 if area is equal across all blocks in the study area, as Method 1 assumes that area is homogeneous across all blocks in the study area while Method 3 allows area to vary across blocks (Table 1). It follows then that measures constructed using Method 1 are relatively close to the measures constructed using Method 3 if area is relatively equal across all blocks in the study area. Measures constructed using Method 2 are equivalent to Method 4 if area is uniform across all parcels within each block, as Method 2 assumes that parcels within each block are equal in size while Method 4 allows parcels within each block to vary in size. Similarly, it follows that measures constructed using Method 2 are relatively close to the measures constructed using Method 4 if parcels within each block are similarly-sized. This has implications when deciding upon construction methods to formulate and perform sensitivity analysis. Researchers should consider the underlying geography of the study area, as this may shed light on observed associations with health outcomes. Further, operationalizing measures for the main analysis and sensitivity analysis using formulations that result in less similar measures (e.g., comparing Methods 2 and 3 or comparing Methods 1 and 4) may provide additional insights about the robustness of the study findings.

Our study contains important limitations. First, we assumed that variables within each index contribute equally, which may influence BE indices if at least one of the variables disproportionately influences the index environment more than other variables (e.g., barbed wire impacts the territoriality index more than security signs). Alternative weighting schemes, for example based on the proportion of events of a variable in a census block, may alter block ranks and consequently findings related to reliability. Second, since there is no precedence for evaluating the impact of construction method on relationships among different indices, we used an exploratory approach based on Spearman’s correlations. However, as research on the implications of methodological choice develops, more rigorous methods that are conducive to the case of simultaneously evaluating multiple indices may emerge. Third, while our categories of strength of preservation are admittedly arbitrary, we sought to be consistent with previous research assessing index reliability. Fourth, we strove to understand the effect of methodological heterogeneity on objective measures of the BE. Measures derived from resident surveys on neighborhood perception (i.e., how individuals perceive their environment), which research suggests may be meaningful to health outcomes [39–41], may result in alternative findings pertaining to reliability. Further, perceptive measures of the BE may more accurately measure the BE that affects a person’s health, as the definition of “neighborhood” is sometimes decided by the interviewee. Fifth, similarities among indices and relationships among indices constructed using Methods 1 and 3, and Methods 2 and 4, are at least partially dependent on characteristics of our study area. Data were collected in a densely populated and urban area, where area was somewhat consistent across blocks in the study area and each block contained similarly-sized parcels. Consequently, certain indices and relationships among indices constructed using Methods 1 and 3, and Methods 2 and 4, were similar. We anticipate that our results are likely robust to other urban areas but less likely to be similar to suburban and rural areas where blocks and parcels within each block are less similar in size. Finally, as data were collected in a mid-sized city located in the southeastern United States, our study may not be generalizable to ultra-dense BEs like Hong Kong, Beijing, or Mumbai [42, 43]. For example, in such environments, standardizing by some measure of dwelling density may be more appropriate than a simple measure of geographic area. Moreover, the reliability of indices in ultra-dense BEs may be especially sensitive to spatial scale due to the presence of increased geographic heterogeneity. More research is needed in this area.

## Conclusions

To our knowledge, this is the first study to respond to researchers’ calls for a systematic assessment of the impact of methodological choice on BE indices in the areas of construction method and spatial scale [12, 14–16]. In particular, using a parcel-level objective survey on the BE, we demonstrate that (1) reliability of BE indices is sensitive to construction method and spatial scale; and (2) given the index-specific variation that we observed, evaluation needs to take place on a case-by-case basis. The underlying geography of the study area, characterized in our study according to the number of parcels and the geographic area, determines whether the potential to observe BE variables is uniform across spatial units. Varying potential for observation, for example according to the number of parcels within census blocks across the study area, translates to increased sensitivity of reliability to construction method. Spatial scale of measurement may be particularly important to reliability in study areas with highly heterogeneous BEs [44, 45]. In the absence of an a priori reason to choose a certain BE construction method, examining alternative constructions of BE indices, while carefully considering methodological assumptions, may provide insight into features of local geography driving analytical findings.

The assessment approach presented here may act as a guide for researchers who seek to investigate the implications of methodological choice in constructing summary indices. In conclusion, we summarize our approach: (1) Identify alternative construction methods based on characteristics of the study area, along with different spatial scales to examine; (2) Assess reliability of each BE index (across construction methods) by determining if alternatively-constructed measures of the same index rank similarly; (3) Assess reliability of relationships among BE indices (within construction method) by examining if pairwise associations are sensitive to construction method; and (4) Examine the influence of spatial scale by replicating steps 2) and 3) with BE indices constructed at alternative spatial scales. Probing the implications of methodological choice in BE construction may help explain inconsistencies in associations between the BE and health.

## Additional files

10.1186/s12942-016-0044-x Community Assessment Project (CAP) variables and PAC, SAC, and 2008 results.
10.1186/s12942-016-0044-x R code for constructing PACs and SACs from a shapefile.

## Abbreviations

BE: built environment
GIS: geographic information system
CAP: Community Assessment Project
PAC: primary adjacency community
SAC: secondary adjacency community
IRR: inter-rater reliability
MAD: mean absolute difference
SD: standard deviation
