# Supplementary material for: Implications of construction method and spatial scale on measures of the built environment
Source: Int J Health Geogr. 2016 Apr 28;15:15. doi: 10.1186/s12942-016-0044-x (PMC4849096; doi:10.1186/s12942-016-0044-x)
Supplement: Supplementary file 1 — 10.1186/s12942-016-0044-x Community Assessment Project (CAP) variables and PAC, SAC, and 2008 results. [file 12942_2016_44_MOESM1_ESM.docx]

**Additional File 1**

**Implications of Construction Method and Spatial Scale on Measures of the Built Environment**

Julie Strominger, Rebecca Anthopolos, and Marie Lynn Miranda

**Table of Contents**

**Table S1.** Community Assessment Project (CAP) variables.

**Table S2.** Summary statistics of the seven built environment indices by method, PAC and SAC levels, 2011 (N=1380).

**Table S3.** Spearman’s correlations between alternatively-constructed measures of each index, PAC and SAC levels, 2011 (N=1380).

**Table S4.** Summary statistics of the mean absolute difference (MAD) in rank for each index, PAC and SAC levels, 2011 (N=1380).

**Table S5.** Spearman’s correlations among indices by method, PAC level, 2011 (N=1380).

**Table S6.** Spearman’s correlations among indices by method, SAC level, 2011 (N=1380).

**Table S7.** Summary statistics of the seven built environment indices by method, census block, PAC, and SAC levels, 2008 (N=886).

**Table S8.** Spearman’s correlations between alternatively-constructed measures of each index, census block, PAC, and SAC levels, 2008 (N=886).

**Table S9.** Summary statistics of the mean absolute difference (MAD) in rank for each index, census block, PAC and SAC levels, 2008 (N=886).

**Table S10.** Spearman’s correlations among indices by method, census block level, 2008 (N=886).

**Table S11.** Spearman’s correlations among indices by method, PAC level, 2008 (N=886).

**Table S12.** Spearman’s correlations among indices by method, SAC level, 2008 (N=886).

**Table S1.** Community Assessment Project (CAP) variables.

| **Housing damage** | **Property disorder** | **Territoriality** | **Vacancy** | **Public nuisances** | **Crime** | **Tenancy** |
| --- | --- | --- | --- | --- | --- | --- |
| Boarded door  Holes in walls  Roof damage  Chimney damage  Foundation damage  Entry damage  Door damage  Peeling paint  Fire damage  Condemned  Boarded windows  Broken windows | Cars on lawn  No grass  Standing water  Litter  Garbage  Broken glass  Discarded furniture  Discarded appliances  Discarded tires  Inoperable vehicle  High weeds  Graffiti  Fencing damage | Security bars  Barbed wire  No trespassing sign  Beware of dog sign  Security sign  Fencing | Vacant parcels | Drug paraphernalia  Graffiti  Food garbage  Shopping carts  Inoperable vehicle  Tree debris  Dog waste  Large trash  Discarded furniture  Batteries  Discarded appliances  Fallen wire  Discarded tires  Broken water meter  Condoms  Uncovered storm drain  Cigarette butts  Baby diapers  Alcohol container  Construction debris  Clothes  Deep holes  Broken glass  Standing water  High weeds | Total crime count | Renter occupied parcels |

**Table S2.** Summary statistics of the seven built environment indices by method, PAC and SAC levels, 2011 (N=1380).

|  |  | **PAC** | |  | **SAC** | |
| --- | --- | --- | --- | --- | --- | --- |
| **Method 1** |  | **Mean (SD)** | **Minimum-Maximum** |  | **Mean (SD)** | **Minimum-Maximum** |
| Housing damage |  | 0 (8.18) | -5.73-52.88 |  | 0 (9.18) | -7.68-44.42 |
| Property disorder |  | 0 (9.94) | -10.5-70.05 |  | 0 (10.59) | -13.72-54.14 |
| Territoriality |  | 0 (4.38) | -5.81-37.37 |  | 0 (4.63) | -7.39-25.66 |
| Vacancy |  | 0 (1.00) | -1.01-7.18 |  | 0 (.001) | -1.28-4.74 |
| Public nuisances |  | 0 (13.66) | -18.7-82.16 |  | 0 (14.98) | -25.27-59.62 |
| Crime |  | 0 (1.00) | -0.89-9.04 |  | 0 (.001) | -1.23-5.17 |
| Tenancy |  | 0 (1.00) | -1.23-9.22 |  | 0 (1.00) | -1.58-5.26 |
| **Method 2** |  | **Mean (SD)** | **Minimum-Maximum** |  | **Mean (SD)** | **Minimum-Maximum** |
| Housing damage |  | 0 (7.71) | -5.90-50.83 |  | 0 (8.90) | -7.68-45.96 |
| Property disorder |  | 0 (9.06) | -12.05-36.18 |  | 0 (10.17) | -13.72-32.45 |
| Territoriality |  | 0 (3.00) | -9.47-23.95 |  | 0 (2.94) | -13.00-10.32 |
| Vacancy |  | 0 (1.00) | -1.09-7.74 |  | 0 (1.00) | -1.25-4.90 |
| Public nuisances |  | 0 (12.63) | -18.26-82.42 |  | 0 (13.07) | -23.42-83.31 |
| Crime^a^ |  | - | - |  | - | - |
| Tenancy |  | 0 (1.00) | -2.05-2.40 |  | 0 (1.00) | -2.12-2.99 |
| **Method 3** |  | **Mean (SD)** | **Minimum-Maximum** |  | **Mean (SD)** | **Minimum-Maximum** |
| Housing damage |  | 0 (7.98) | -5.06-46.51 |  | 0 (9.16) | -6.67-41.55 |
| Property disorder |  | 0 (9.94) | -9.70-52.21 |  | 0 (10.90) | -11.84-37.45 |
| Territoriality |  | 0 (4.09) | -6.19-18.02 |  | 0 (4.46) | -7.97-16.43 |
| Vacancy |  | 0 (1.00) | -0.80-7.68 |  | 0 (1.00) | -0.93-5.84 |
| Public nuisances |  | 0 (12.79) | -17.97-79.33 |  | 0 (14.8) | -19.4-63.92 |
| Crime |  | 0 (1.00) | -1.16-8.73 |  | 0 (1.00) | -1.26-6.33 |
| Tenancy |  | 0 (1.00) | -1.44-3.49 |  | 0 (1.00) | -1.39-3.26 |
| **Method 4** |  | **Mean (SD)** | **Minimum-Maximum** |  | **Mean (SD)** | **Minimum-Maximum** |
| Housing damage |  | 0 (6.25) | -4.13-47.62 |  | 0 (7.31) | -5.89-29.56 |
| Property disorder |  | 0 (7.29) | -9.35-33.7 |  | 0 (8.26) | -11.39-34.97 |
| Territoriality |  | 0 (3.25) | -7.21-20.19 |  | 0 (3.36) | -10.23-15.63 |
| Vacancy |  | 0 (1.00) | -1.16-5.66 |  | 0 (1.00) | -1.48-4.33 |
| Public nuisances |  | 0 (10.70) | -15.41-55.41 |  | 0 (12.01) | -18.38-58.17 |
| Crime^a^ |  | - | - |  | - | - |
| Tenancy |  | 0 (1.00) | -2.73-1.83 |  | 0 (.001) | -3.29-2.40 |

Abbreviations: SD, standard deviation; PAC, primary adjacency community; SAC, secondary adjacency community.

Method 1 is a simple count, Method 2 is an average count per parcel, Method 3 is an average count per unit area, and Method 4 is proportion of area with a variable present.

^a^Crime is not constructed using Methods 2 or 4 as crime is measured at the block level.

**Table S3.** Spearman’s correlations between alternatively-constructed measures of each index, PAC and SAC levels, 2011 (N=1380).

|  |  | **PAC** | | | |  | **SAC** | | | |
| --- | --- | --- | --- | --- | --- | --- | --- | --- | --- | --- |
|  |  | **(1)** | **(2)** | **(3)** | **(4)** |  | **(1)** | **(2)** | **(3)** | **(4)** |
| **Housing damage** |  |  |  |  |  |  |  |  |  |  |
| Method 1 (1) |  | 1.00 | 0.88 | 0.86 | 0.83 |  | 1.00 | 0.86 | 0.85 | 0.85 |
| Method 2 (2) |  |  | 1.00 | 0.95 | 0.92 |  |  | 1.00 | 0.97 | 0.94 |
| Method 3 (3) |  |  |  | 1.00 | 0.91 |  |  |  | 1.00 | 0.93 |
| Method 4 (4) |  |  |  |  | 1.00 |  |  |  |  | 1.00 |
| **Property disorder** |  |  |  |  |  |  |  |  |  |  |
| Method 1 |  | 1.00 | 0.77 | 0.72 | 0.73 |  | 1.00 | 0.72 | 0.68 | 0.73 |
| Method 2 |  |  | 1.00 | 0.90 | 0.90 |  |  | 1.00 | 0.93 | 0.93 |
| Method 3 |  |  |  | 1.00 | 0.86 |  |  |  | 1.00 | 0.91 |
| Method 4 |  |  |  |  | 1.00 |  |  |  |  | 1.00 |
| **Territoriality** |  |  |  |  |  |  |  |  |  |  |
| Method 1 |  | 1.00 | 0.49 | 0.35 | 0.30 |  | 1.00 | 0.44 | 0.25 | 0.28 |
| Method 2 |  |  | 1.00 | 0.72 | 0.72 |  |  | 1.00 | 0.75 | 0.79 |
| Method 3 |  |  |  | 1.00 | 0.68 |  |  |  | 1.00 | 0.77 |
| Method 4 |  |  |  |  | 1.00 |  |  |  |  | 1.00 |
| **Vacancy** |  |  |  |  |  |  |  |  |  |  |
| Method 1 |  | 1.00 | 0.72 | 0.65 | 0.70 |  | 1.00 | 0.67 | 0.57 | 0.70 |
| Method 2 |  |  | 1.00 | 0.84 | 0.70 |  |  | 1.00 | 0.88 | 0.67 |
| Method 3 |  |  |  | 1.00 | 0.58 |  |  |  | 1.00 | 0.54 |
| Method 4 |  |  |  |  | 1.00 |  |  |  |  | 1.00 |
| **Public nuisances** |  |  |  |  |  |  |  |  |  |  |
| Method 1 |  | 1.00 | 0.87 | 0.65 | 0.44 |  | 1.00 | 0.79 | 0.58 | 0.39 |
| Method 2 |  |  | 1.00 | 0.65 | 0.43 |  |  | 1.00 | 0.71 | 0.50 |
| Method 3 |  |  |  | 1.00 | 0.68 |  |  |  | 1.00 | 0.76 |
| Method 4 |  |  |  |  | 1.00 |  |  |  |  | 1.00 |
| **Crime^a^** |  |  |  |  |  |  |  |  |  |  |
| Method 1 |  | 1.00 | - | 0.45 | - |  | 1.00 | - | 0.29 | - |
| Method 3 |  |  |  | 1.00 | - |  |  |  | 1.00 | - |
| **Tenancy** |  |  |  |  |  |  |  |  |  |  |
| Method 1 |  | 1.00 | 0.19 | 0.20 | 0.19 |  | 1.00 | 0.08 | 0.02 | 0.13 |
| Method 2 |  |  | 1.00 | 0.62 | 0.79 |  |  | 1.00 | 0.70 | 0.80 |
| Method 3 |  |  |  | 1.00 | 0.19 |  |  |  | 1.00 | 0.27 |
| Method 4 |  |  |  |  | 1.00 |  |  |  |  | 1.00 |

Abbreviations: PAC, primary adjacency community; SAC, secondary adjacency community.

Method 1 is a simple count, Method 2 is an average count per parcel, Method 3 is an average count per unit area, and Method 4 is proportion of area with a variable present.

^a^Crime is not constructed using Methods 2 or 4 as crime is measured at the block level.

**Table S4.** Summary statistics of the mean absolute difference (MAD) in rank for each index, PAC and SAC levels, 2011 (N=1380).

|  | **PAC** |  | **SAC** |
| --- | --- | --- | --- |
| **Index** | **Mean (SD)** |  | **Mean (SD)** |
| Housing damage | 79.45 (56.92) |  | 77.46 (53.36) |
| Property disorder | 110.13 (66.53) |  | 107.17 (68.06) |
| Territoriality | 178.58 (95.06) |  | 179.61 (87.43) |
| Vacancy | 141.59 (87.57) |  | 151.3 (87.86) |
| Public nuisances | 163.12 (100.06) |  | 160.72 (97.89) |
| Crime^a^ | 166.66 (127.26) |  | 194.13 (136.79) |
| Tenancy | 214.47 (115.15) |  | 219.35 (111.72) |

Abbreviations: SD, standard deviation; PAC, primary adjacency community; SAC, secondary adjacency community.

^a^Crime is not constructed using Methods 2 or 4 as crime is measured at the block level.

**Table S5.** Spearman’s correlations among indices by method, PAC level, 2011 (N=1380).

|  | **Housing damage** | **Property disorder** | **Territoriality** | **Vacancy** | **Public nuisances** | **Crime** | **Tenancy** |
| --- | --- | --- | --- | --- | --- | --- | --- |
| **Method 1** |  |  |  |  |  |  |  |
| Housing damage | 1.00 | 0.81 | 0.62 | 0.57 | 0.74 | 0.40 | 0.52 |
| Property disorder |  | 1.00 | 0.82 | 0.75 | 0.87 | 0.58 | 0.73 |
| Territoriality |  |  | 1.00 | 0.74 | 0.80 | 0.64 | 0.82 |
| Vacancy |  |  |  | 1.00 | 0.74 | 0.53 | 0.74 |
| Public nuisances |  |  |  |  | 1.00 | 0.60 | 0.77 |
| Crime |  |  |  |  |  | 1.00 | 0.65 |
| Tenancy |  |  |  |  |  |  | 1.00 |
| **Method 2** |  |  |  |  |  |  |  |
| Housing damage | 1.00 | 0.79 | 0.57 | 0.49 | 0.44 | - | 0.59 |
| Property disorder |  | 1.00 | 0.63 | 0.63 | 0.56 | - | 0.68 |
| Territoriality |  |  | 1.00 | 0.31 | 0.36 | - | 0.25 |
| Vacancy |  |  |  | 1.00 | 0.43 | - | 0.64 |
| Public nuisances |  |  |  |  | 1.00 | - | 0.37 |
| Crime^a^ |  |  |  |  |  | - | - |
| Tenancy |  |  |  |  |  |  | 1.00 |
| **Method 3** |  |  |  |  |  |  |  |
| Housing damage | 1.00 | 0.88 | 0.81 | 0.69 | 0.81 | 0.63 | 0.81 |
| Property disorder |  | 1.00 | 0.85 | 0.79 | 0.84 | 0.64 | 0.85 |
| Territoriality |  |  | 1.00 | 0.66 | 0.72 | 0.54 | 0.80 |
| Vacancy |  |  |  | 1.00 | 0.72 | 0.49 | 0.72 |
| Public nuisances |  |  |  |  | 1.00 | 0.64 | 0.80 |
| Crime |  |  |  |  |  | 1.00 | 0.63 |
| Tenancy |  |  |  |  |  |  | 1.00 |
| **Method 4** |  |  |  |  |  |  |  |
| Housing damage | 1.00 | 0.75 | 0.60 | 0.15 | 0.55 | - | 0.22 |
| Property disorder |  | 1.00 | 0.55 | 0.29 | 0.55 | - | 0.30 |
| Territoriality |  |  | 1.00 | 0.02 | 0.42 | - | 0.01 |
| Vacancy |  |  |  | 1.00 | 0.12 | - | 0.30 |
| Public nuisances |  |  |  |  | 1.00 | - | 0.58 |
| Crime^a^ |  |  |  |  |  | - | - |
| Tenancy |  |  |  |  |  |  | 1.00 |

Abbreviations: PAC, primary adjacency community.

Method 1 is a simple count, Method 2 is an average count per parcel, Method 3 is an average count per unit area, and Method 4 is proportion of area with a variable present.

^a^Crime is not constructed using Methods 2 or 4 as crime is measured at the block level.

**Table S6.** Spearman’s correlations among indices by method, SAC level, 2011 (N=1380).

|  | **Housing damage** | **Property disorder** | **Territoriality** | **Vacancy** | **Public nuisances** | **Crime** | **Tenancy** |
| --- | --- | --- | --- | --- | --- | --- | --- |
| **Method 1** |  |  |  |  |  |  |  |
| Housing damage | 1.00 | 0.86 | 0.63 | 0.64 | 0.81 | 0.38 | 0.48 |
| Property disorder |  | 1.00 | 0.85 | 0.80 | 0.93 | 0.58 | 0.71 |
| Territoriality |  |  | 1.00 | 0.82 | 0.85 | 0.70 | 0.88 |
| Vacancy |  |  |  | 1.00 | 0.80 | 0.54 | 0.75 |
| Public nuisances |  |  |  |  | 1.00 | 0.63 | 0.77 |
| Crime |  |  |  |  |  | 1.00 | 0.72 |
| Tenancy |  |  |  |  |  |  | 1.00 |
| **Method 2** |  |  |  |  |  |  |  |
| Housing damage | 1.00 | 0.87 | 0.69 | 0.64 | 0.56 | - | 0.70 |
| Property disorder |  | 1.00 | 0.76 | 0.72 | 0.70 | - | 0.79 |
| Territoriality |  |  | 1.00 | 0.52 | 0.57 | - | 0.48 |
| Vacancy |  |  |  | 1.00 | 0.57 | - | 0.73 |
| Public nuisances |  |  |  |  | 1.00 | - | 0.57 |
| Crime^a^ |  |  |  |  |  | - | - |
| Tenancy |  |  |  |  |  |  | 1.00 |
| **Method 3** |  |  |  |  |  |  |  |
| Housing damage | 1.00 | 0.93 | 0.88 | 0.81 | 0.89 | 0.70 | 0.85 |
| Property disorder |  | 1.00 | 0.93 | 0.86 | 0.92 | 0.71 | 0.90 |
| Territoriality |  |  | 1.00 | 0.78 | 0.87 | 0.64 | 0.89 |
| Vacancy |  |  |  | 1.00 | 0.81 | 0.58 | 0.79 |
| Public nuisances |  |  |  |  | 1.00 | 0.71 | 0.87 |
| Crime |  |  |  |  |  | 1.00 | 0.73 |
| Tenancy |  |  |  |  |  |  | 1.00 |
| **Method 4** |  |  |  |  |  |  |  |
| Housing damage | 1.00 | 0.84 | 0.73 | 0.22 | 0.64 | - | 0.27 |
| Property disorder |  | 1.00 | 0.71 | 0.34 | 0.64 | - | 0.37 |
| Territoriality |  |  | 1.00 | 0.19 | 0.58 | - | 0.14 |
| Vacancy |  |  |  | 1.00 | 0.11 | - | 0.33 |
| Public nuisances |  |  |  |  | 1.00 | - | 0.62 |
| Crime^a^ |  |  |  |  |  | - | - |
| Tenancy |  |  |  |  |  |  | 1.00 |

Abbreviations: SAC, secondary adjacency community.

Method 1 is a simple count, Method 2 is an average count per parcel, Method 3 is an average count per unit area, and Method 4 is proportion of area with a variable present.

^a^Crime is not constructed using Methods 2 or 4 as crime is measured at the block level.

**Table S7.** Summary statistics of the seven built environment indices by method, census block, PAC, and SAC levels, 2008 (N=886).

|  |  | **Block** | |  | **PAC** | |  | **SAC** |  |
| --- | --- | --- | --- | --- | --- | --- | --- | --- | --- |
| **Method 1** |  | **Mean (SD)** | **Minimum-Maximum** |  | **Mean (SD)** | **Minimum-Maximum** |  | **Mean (SD)** | **Minimum-Maximum** |
| Housing damage |  | 0 (7.00) | -4.44-69.27 |  | 0 (8.71) | -8.87-42.81 |  | 0 (9.72) | -11.91-34.15 |
| Property disorder |  | 0 (8.58) | -6.63-82.03 |  | 0 (10.24) | -12.04-61.95 |  | 0 (10.99) | -15.17-54.22 |
| Territoriality |  | 0 (3.89) | -3.82-24.04 |  | 0 (4.11) | -6.61-23.24 |  | 0 (4.29) | -8.25-19.27 |
| Vacancy^a^ |  | 0 (1.00) | -0.71-10.19 |  | 0 (1.00) | -1.18-5.45 |  | 0 (1.00) | -1.38-4.43 |
| Public nuisances^b^ |  | 0 (11.00) | -9.97-81.41 |  | 0 (14.45) | -19.03-83.07 |  | 0 (16.81) | -25.49-78.32 |
| Crime |  | 0 (1.00) | -0.53-18.64 |  | 0 (1.00) | -0.98-8.07 |  | 0 (1.00) | -1.30-5.36 |
| Tenancy^c^ |  | 0 (1.00) | -0.97-8.07 |  | 0 (1.00) | -1.55-5.58 |  | 0 (1.00) | -1.92-4.09 |
| **Method 2** |  | **Mean (SD)** | **Minimum-Maximum** |  | **Mean (SD)** | **Minimum-Maximum** |  | **Mean (SD)** | **Minimum-Maximum** |
| Housing damage |  | 0 (6.00) | -4.19-57.08 |  | 0 (7.93) | -9.59-58.52 |  | 0 (9.07) | -13.70-31.14 |
| Property disorder |  | 0 (6.27) | -6.27-42.12 |  | 0 (8.62) | -13.87-41.00 |  | 0 (9.61) | -18.30-39.37 |
| Territoriality |  | 0 (2.86) | -4.40-23.14 |  | 0 (2.65) | -9.55-13.96 |  | 0 (2.51) | -12.57-18.46 |
| Vacancy |  | 0 (1.00) | -0.81-3.95 |  | 0 (1.00) | -1.35-4.69 |  | 0 (1.00) | -1.48-3.36 |
| Public nuisances |  | 0 (10.00) | -8.45-189.48 |  | 0 (13.22) | -19.36-71.8 |  | 0 (14.59) | -24.45-47.64 |
| Crime^d^ |  | - | - |  | - | - |  | - | - |
| Tenancy |  | 0 (1.00) | -2.18-1.41 |  | 0 (1.00) | -2.32-2.18 |  | 0 (1.00) | -2.52-2.63 |
| **Method 3** |  | **Mean (SD)** | **Minimum-Maximum** |  | **Mean (SD)** | **Minimum-Maximum** |  | **Mean (SD)** | **Minimum-Maximum** |
| Housing damage |  | 0 (6.43) | -4.05-49.29 |  | 0 (8.51) | -8.65-47.95 |  | 0 (9.44) | -12.09-31.70 |
| Property disorder |  | 0 (6.76) | -5.85-43.82 |  | 0 (9.47) | -11.96-47.21 |  | 0 (10.10) | -15.81-30.05 |
| Territoriality |  | 0 (3.12) | -3.91-22.54 |  | 0 (3.60) | -7.16-12.19 |  | 0 (3.73) | -9.35-12.05 |
| Vacancy |  | 0 (1.00) | -0.64-9.60 |  | 0 (1.00) | -1.09-5.64 |  | 0 (1.00) | -1.29-3.47 |
| Public nuisances |  | 0 (8.55) | -9.33-56.4 |  | 0 (11.88) | -19.76-46.01 |  | 0 (13.64) | -24.48-36.63 |
| Crime |  | 0 (1.00) | -0.75-13.46 |  | 0 (1.00) | -1.42-5.35 |  | 0 (1.00) | -1.58-3.74 |
| Tenancy |  | 0 (1.00) | -0.99-16.28 |  | 0 (1.00) | -1.67-3.15 |  | 0 (1.00) | -1.92-2.81 |
| **Method 4** |  | **Mean (SD)** | **Minimum-Maximum** |  | **Mean (SD)** | **Minimum-Maximum** |  | **Mean (SD)** | **Minimum-Maximum** |
| Housing damage |  | 0 (5.97) | -3.83-52.18 |  | 0 (7.03) | -7.25-38.93 |  | 0 (8.03) | -10.23-26.68 |
| Property disorder |  | 0 (5.89) | -5.73-40.18 |  | 0 (7.20) | -10.44-33.98 |  | 0 (8.04) | -13.96-26.20 |
| Territoriality |  | 0 (2.89) | -4.18-18.93 |  | 0 (3.15) | -7.4-16.87 |  | 0 (3.22) | -9.88-12.30 |
| Vacancy |  | 0 (1.00) | -0.76-3.91 |  | 0 (1.00) | -1.35-4.56 |  | 0 (1.00) | -1.71-3.93 |
| Public nuisances |  | 0 (9.5) | -9.06-158.77 |  | 0 (10.24) | -16.42-81.92 |  | 0 (11.51) | -21.43-69.81 |
| Crime^d^ |  | - | - |  | - | - |  | - | - |
| Tenancy |  | 0 (1.00) | -2.16-1.30 |  | 0 (1.00) | -2.92-1.74 |  | 0 (1.00) | -3.24-2.14 |

Abbreviations: SD, standard deviation; PAC, primary adjacency community; SAC, secondary adjacency community.

Method 1 is a simple count, Method 2 is an average count per parcel, Method 3 is an average count per unit area, and Method 4 is proportion of area with a variable present.

^a^N for vacancy at the block level is 884 due to data availability.

^b^N for public nuisances at the block level is 876, N for public nuisances at the PAC level is 883, and N for public nuisances at the SAC level is 885 due to data availability.

^c^N for tenancy at the block level is 877 due to data availability.

^d^Crime is not constructed using Methods 2 or 4 as crime is measured at the block level.

**Table S8.** Spearman’s correlations between alternatively-constructed measures of each index, census block, PAC, and SAC levels, 2008 (N=886).

|  |  | **Block** | | | |  | **PAC** | | | |  | **SAC** | | | |
| --- | --- | --- | --- | --- | --- | --- | --- | --- | --- | --- | --- | --- | --- | --- | --- |
|  |  | **(1)** | **(2)** | **(3)** | **(4)** |  | **(1)** | **(2)** | **(3)** | **(4)** |  | **(1)** | **(2)** | **(3)** | **(4)** |
| **Housing damage** |  |  |  |  |  |  |  |  |  |  |  |  |  |  |  |
| Method 1 (1) |  | 1.00 | 0.93 | 0.92 | 0.90 |  | 1.00 | 0.88 | 0.86 | 0.84 |  | 1.00 | 0.86 | 0.83 | 0.83 |
| Method 2 (2) |  |  | 1.00 | 0.96 | 0.97 |  |  | 1.00 | 0.93 | 0.91 |  |  | 1.00 | 0.94 | 0.93 |
| Method 3 (3) |  |  |  | 1.00 | 0.95 |  |  |  | 1.00 | 0.92 |  |  |  | 1.00 | 0.93 |
| Method 4 (4) |  |  |  |  | 1.00 |  |  |  |  | 1.00 |  |  |  |  | 1.00 |
| **Property disorder** |  |  |  |  |  |  |  |  |  |  |  |  |  |  |  |
| Method 1 |  | 1.00 | 0.83 | 0.82 | 0.80 |  | 1.00 | 0.80 | 0.79 | 0.79 |  | 1.00 | 0.80 | 0.77 | 0.81 |
| Method 2 |  |  | 1.00 | 0.88 | 0.95 |  |  | 1.00 | 0.87 | 0.92 |  |  | 1.00 | 0.91 | 0.93 |
| Method 3 |  |  |  | 1.00 | 0.85 |  |  |  | 1.00 | 0.81 |  |  |  | 1.00 | 0.85 |
| Method 4 |  |  |  |  | 1.00 |  |  |  |  | 1.00 |  |  |  |  | 1.00 |
| **Territoriality** |  |  |  |  |  |  |  |  |  |  |  |  |  |  |  |
| Method 1 |  | 1.00 | 0.66 | 0.64 | 0.60 |  | 1.00 | 0.55 | 0.48 | 0.38 |  | 1.00 | 0.60 | 0.46 | 0.36 |
| Method 2 |  |  | 1.00 | 0.72 | 0.91 |  |  | 1.00 | 0.59 | 0.73 |  |  | 1.00 | 0.58 | 0.70 |
| Method 3 |  |  |  | 1.00 | 0.68 |  |  |  | 1.00 | 0.60 |  |  |  | 1.00 | 0.64 |
| Method 4 |  |  |  |  | 1.00 |  |  |  |  | 1.00 |  |  |  |  | 1.00 |
| **Vacancy^a^** |  |  |  |  |  |  |  |  |  |  |  |  |  |  |  |
| Method 1 |  | 1.00 | 0.85 | 0.86 | 0.84 |  | 1.00 | 0.77 | 0.80 | 0.69 |  | 1.00 | 0.78 | 0. 80 | 0.69 |
| Method 2 |  |  | 1.00 | 0.92 | 0.94 |  |  | 1.00 | 0.87 | 0.76 |  |  | 1.00 | 0.91 | 0.81 |
| Method 3 |  |  |  | 1.00 | 0.90 |  |  |  | 1.00 | 0.69 |  |  |  | 1.00 | 0.71 |
| Method 4 |  |  |  |  | 1.00 |  |  |  |  | 1.00 |  |  |  |  | 1.00 |
| **Public nuisances^b^** |  |  |  |  |  |  |  |  |  |  |  |  |  |  |  |
| Method 1 |  | 1.00 | 0.62 | 0.86 | 0.62 |  | 1.00 | 0.91 | 0.81 | 0.62 |  | 1.00 | 0.88 | 0.78 | 0.64 |
| Method 2 |  |  | 1.00 | 0.74 | 0.94 |  |  | 1.00 | 0.83 | 0.68 |  |  | 1.00 | 0.85 | 0.77 |
| Method 3 |  |  |  | 1.00 | 0.70 |  |  |  | 1.00 | 0.69 |  |  |  | 1.00 | 0.79 |
| Method 4 |  |  |  |  | 1.00 |  |  |  |  | 1.00 |  |  |  |  | 1.00 |
| **Crime^c^** |  |  |  |  |  |  |  |  |  |  |  |  |  |  |  |
| Method 1 |  | 1.00 |  | 0.81 |  |  | 1.00 |  | 0.61 |  |  | 1.00 |  | 0.53 |  |
| Method 3 |  |  |  | 1.00 |  |  |  |  | 1.00 |  |  |  |  | 1.00 |  |
| **Tenancy^d^** |  |  |  |  |  |  |  |  |  |  |  |  |  |  |  |
| Method 1 |  | 1.00 | 0.23 | 0.49 | 0.22 |  | 1.00 | 0.32 | 0.39 | 0.19 |  | 1.00 | 0.29 | 0.32 | 0.25 |
| Method 2 |  |  | 1.00 | 0.46 | 0.94 |  |  | 1.00 | 0.58 | 0.85 |  |  | 1.00 | 0.66 | 0.89 |
| Method 3 |  |  |  | 1.00 | 0.36 |  |  |  | 1.00 | 0.24 |  |  |  | 1.00 | 0.36 |
| Method 4 |  |  |  |  | 1.00 |  |  |  |  | 1.00 |  |  |  |  | 1.00 |

Abbreviations: PAC, primary adjacency community; SAC, secondary adjacency community.

Method 1 is a simple count, Method 2 is an average count per parcel, Method 3 is an average count per unit area, and Method 4 is proportion of area with a variable present.

^a^N for vacancy at the block level is 884 due to data availability.

^b^N for public nuisances at the block level is 876, N for public nuisances at the PAC level is 883, and N for public nuisances at the SAC level is 885 due to data availability.

^c^Crime is not constructed using Methods 2 or 4 as crime is measured at the block level.

^d^N for tenancy at the block level is 877 due to data availability.

**Table S9.** Summary statistics of the mean absolute difference (MAD) in rank for each index, census block, PAC and SAC levels, 2008 (N=886).

|  | **Block** |  | **PAC** |  | **SAC** |
| --- | --- | --- | --- | --- | --- |
| **Index** | **Mean (SD)** |  | **Mean (SD)** |  | **Mean (SD)** |
| Housing damage | 33.44 (33.77) |  | 52.91 (36.14) |  | 53.3 (36.18) |
| Property disorder | 56.72 (48.5) |  | 65.92 (43.59) |  | 61.65 (42.1) |
| Territoriality | 86.72 (65.71) |  | 113.23 (64.82) |  | 113.12 (64.52) |
| Vacancy^a^ | 44.21 (48.59) |  | 78.22 (50.94) |  | 74.34 (49.35) |
| Public nuisances^b^ | 79.11 (64.05) |  | 81.44 (48.79) |  | 76.02 (46.82) |
| Crime | 52.47 (59.01) |  | 84.64 (75.45) |  | 94.52 (79.8) |
| Tenancy^c^ | 122.07 (85.41) |  | 128.38 (74.74) |  | 122.67 (75.33) |

Abbreviations: SD, standard deviation; PAC, primary adjacency community; SAC, secondary adjacency community.

^a^N for vacancy at the block level is 884 due to data availability.

^b^N for public nuisances at the block level is 876, N for public nuisances at the PAC level is 883, and N for public nuisances at the SAC level is 885 due to data availability.

^c^N for tenancy at the block level is 877 due to data availability.

**Table S10.** Spearman’s correlations among indices by method, census block level, 2008 (N=886).

|  | **Housing damage** | **Property disorder** | **Territoriality** | **Vacancy^a^** | **Public nuisances^b^** | **Crime** | **Tenancy^c^** |
| --- | --- | --- | --- | --- | --- | --- | --- |
| **Method 1** |  |  |  |  |  |  |  |
| Housing damage | 1.00 | 0.75 | 0.59 | 0.61 | 0.63 | 0.40 | 0.58 |
| Property disorder |  | 1.00 | 0.65 | 0.63 | 0.70 | 0.49 | 0.69 |
| Territoriality |  |  | 1.00 | 0.44 | 0.52 | 0.45 | 0.62 |
| Vacancy |  |  |  | 1.00 | 0.60 | 0.38 | 0.65 |
| Public nuisances |  |  |  |  | 1.00 | 0.50 | 0.63 |
| Crime |  |  |  |  |  | 1.00 | 0.55 |
| Tenancy |  |  |  |  |  |  | 1.00 |
| **Method 2** |  |  |  |  |  |  |  |
| Housing damage | 1.00 | 0.60 | 0.29 | 0.43 | 0.28 | - | 0.16 |
| Property disorder |  | 1.00 | 0.23 | 0.41 | 0.43 | - | 0.29 |
| Territoriality |  |  | 1.00 | -0.05 | -0.02 | - | -0.23 |
| Vacancy |  |  |  | 1.00 | 0.34 | - | 0.39 |
| Public nuisances |  |  |  |  | 1.00 | - | 0.57 |
| Crime^d^ |  |  |  |  |  | - | - |
| Tenancy |  |  |  |  |  |  | 1.00 |
| **Method 3** |  |  |  |  |  |  |  |
| Housing damage | 1.00 | 0.72 | 0.56 | 0.55 | 0.56 | 0.39 | 0.57 |
| Property disorder |  | 1.00 | 0.53 | 0.55 | 0.61 | 0.42 | 0.62 |
| Territoriality |  |  | 1.00 | 0.26 | 0.30 | 0.31 | 0.41 |
| Vacancy |  |  |  | 1.00 | 0.51 | 0.27 | 0.63 |
| Public nuisances |  |  |  |  | 1.00 | 0.41 | 0.57 |
| Crime |  |  |  |  |  | 1.00 | 0.43 |
| Tenancy |  |  |  |  |  |  | 1.00 |
| **Method 4** |  |  |  |  |  |  |  |
| Housing damage | 1.00 | 0.57 | 0.28 | 0.41 | 0.25 | - | 0.09 |
| Property disorder |  | 1.00 | 0.23 | 0.41 | 0.39 | - | 0.24 |
| Territoriality |  |  | 1.00 | -0.06 | 0.01 | - | -0.20 |
| Vacancy |  |  |  | 1.00 | 0.29 | - | 0.34 |
| Public nuisances |  |  |  |  | 1.00 | - | 0.53 |
| Crime^d^ |  |  |  |  |  | - | - |
| Tenancy |  |  |  |  |  |  | 1.00 |

Method 1 is a simple count, Method 2 is an average count per parcel, Method 3 is an average count per unit area, and Method 4 is proportion of area with a variable present.

^a^N for pairwise comparisons at the block level between vacancy and housing damage, property disorder, territoriality, or crime is 884, N for the pairwise comparison between vacancy and public nuisances in 874, and N for the pairwise comparison between vacancy and tenancy is 875 due to data availability.

^b^N for pairwise comparisons at the block level between public nuisances and housing damage, property disorder, territoriality, or crime is 876, N for the pairwise comparison between public nuisances and vacancy is 874, and N for the pairwise comparison between public nuisances and tenancy is 875 due to data availability.

^c^N for pairwise comparisons at the block level between tenancy and housing damage, property disorder, territoriality, or crime is 877, N for the pairwise comparison between tenancy and vacancy is 875, and N for the pairwise comparison between tenancy and public nuisances is 868 due to data availability.

^d^Crime is not constructed using Methods 2 or 4 as crime is measured at the block level.

**Table S11.** Spearman’s correlations among indices by method, PAC level, 2008 (N=886).

|  | **Housing damage** | **Property disorder** | **Territoriality** | **Vacancy** | **Public nuisances^a^** | **Crime** | **Tenancy** |
| --- | --- | --- | --- | --- | --- | --- | --- |
| **Method 1** |  |  |  |  |  |  |  |
| Housing damage | 1.00 | 0.83 | 0.72 | 0.80 | 0.81 | 0.57 | 0.69 |
| Property disorder |  | 1.00 | 0.85 | 0.81 | 0.88 | 0.69 | 0.79 |
| Territoriality |  |  | 1.00 | 0.71 | 0.79 | 0.64 | 0.80 |
| Vacancy |  |  |  | 1.00 | 0.79 | 0.62 | 0.75 |
| Public nuisances |  |  |  |  | 1.00 | 0.74 | 0.77 |
| Crime |  |  |  |  |  | 1.00 | 0.68 |
| Tenancy |  |  |  |  |  |  | 1.00 |
| **Method 2** |  |  |  |  |  |  |  |
| Housing damage | 1.00 | 0.75 | 0.46 | 0.67 | 0.60 | - | 0.61 |
| Property disorder |  | 1.00 | 0.50 | 0.67 | 0.71 | - | 0.56 |
| Territoriality |  |  | 1.00 | 0.27 | 0.36 | - | 0.08 |
| Vacancy |  |  |  | 1.00 | 0.51 | - | 0.73 |
| Public nuisances |  |  |  |  | 1.00 | - | 0.48 |
| Crime^b^ |  |  |  |  |  | - | - |
| Tenancy |  |  |  |  |  |  | 1.00 |
| **Method 3** |  |  |  |  |  |  |  |
| Housing damage | 1.00 | 0.83 | 0.76 | 0.79 | 0.8 | 0.64 | 0.78 |
| Property disorder |  | 1.00 | 0.78 | 0.80 | 0.82 | 0.63 | 0.76 |
| Territoriality |  |  | 1.00 | 0.64 | 0.64 | 0.55 | 0.72 |
| Vacancy |  |  |  | 1.00 | 0.74 | 0.60 | 0.80 |
| Public nuisances |  |  |  |  | 1.00 | 0.69 | 0.69 |
| Crime |  |  |  |  |  | 1.00 | 0.69 |
| Tenancy |  |  |  |  |  |  | 1.00 |
| **Method 4** |  |  |  |  |  |  |  |
| Housing damage | 1.00 | 0.71 | 0.42 | 0.52 | 0.51 | - | 0.30 |
| Property disorder |  | 1.00 | 0.41 | 0.55 | 0.60 | - | 0.35 |
| Territoriality |  |  | 1.00 | 0.06 | 0.19 | - | -0.07 |
| Vacancy |  |  |  | 1.00 | 0.45 | - | 0.46 |
| Public nuisances |  |  |  |  | 1.00 | - | 0.62 |
| Crime^b^ |  |  |  |  |  | - | - |
| Tenancy |  |  |  |  |  |  | 1.00 |

Abbreviations: PAC, primary adjacency community.

Method 1 is a simple count, Method 2 is an average count per parcel, Method 3 is an average count per unit area, and Method 4 is proportion of area with a variable present.

^a^N for pairwise comparisons involving public nuisances is 883 due to data availability.

^b^Crime is not constructed using Methods 2 or 4 as crime is measured at the block level.

**Table S12.** Spearman’s correlations among indices by method, SAC level, 2008 (N=886).

|  | **Housing damage** | **Property disorder** | **Territoriality** | **Vacancy** | **Public nuisances^a^** | **Crime** | **Tenancy** |
| --- | --- | --- | --- | --- | --- | --- | --- |
| **Method 1** |  |  |  |  |  |  |  |
| Housing damage | 1.00 | 0.88 | 0.81 | 0.91 | 0.89 | 0.72 | 0.77 |
| Property disorder |  | 1.00 | 0.92 | 0.86 | 0.93 | 0.82 | 0.85 |
| Territoriality |  |  | 1.00 | 0.79 | 0.87 | 0.79 | 0.87 |
| Vacancy |  |  |  | 1.00 | 0.87 | 0.75 | 0.78 |
| Public nuisances |  |  |  |  | 1.00 | 0.86 | 0.87 |
| Crime |  |  |  |  |  | 1.00 | 0.81 |
| Tenancy |  |  |  |  |  |  | 1.00 |
| **Method 2** |  |  |  |  |  |  |  |
| Housing damage | 1.00 | 0.82 | 0.61 | 0.82 | 0.69 | - | 0.74 |
| Property disorder |  | 1.00 | 0.71 | 0.76 | 0.79 | - | 0.65 |
| Territoriality |  |  | 1.00 | 0.46 | 0.56 | - | 0.27 |
| Vacancy |  |  |  | 1.00 | 0.59 | - | 0.84 |
| Public nuisances |  |  |  |  | 1.00 | - | 0.57 |
| Crime^b^ |  |  |  |  |  | - | - |
| Tenancy |  |  |  |  |  |  | 1.00 |
| **Method 3** |  |  |  |  |  |  |  |
| Housing damage | 1.00 | 0.87 | 0.84 | 0.88 | 0.87 | 0.75 | 0.84 |
| Property disorder |  | 1.00 | 0.88 | 0.85 | 0.89 | 0.72 | 0.78 |
| Territoriality |  |  | 1.00 | 0.77 | 0.79 | 0.65 | 0.79 |
| Vacancy |  |  |  | 1.00 | 0.84 | 0.71 | 0.84 |
| Public nuisances |  |  |  |  | 1.00 | 0.77 | 0.74 |
| Crime |  |  |  |  |  | 1.00 | 0.75 |
| Tenancy |  |  |  |  |  |  | 1.00 |
| **Method 4** |  |  |  |  |  |  |  |
| Housing damage | 1.00 | 0.80 | 0.54 | 0.64 | 0.67 | - | 0.50 |
| Property disorder |  | 1.00 | 0.53 | 0.66 | 0.71 | - | 0.46 |
| Territoriality |  |  | 1.00 | 0.15 | 0.33 | - | 0.06 |
| Vacancy |  |  |  | 1.00 | 0.62 | - | 0.63 |
| Public nuisances |  |  |  |  | 1.00 | - | 0.77 |
| Crime^b^ |  |  |  |  |  | - | - |
| Tenancy |  |  |  |  |  |  | 1.00 |

Abbreviations: SAC, secondary adjacency community.

Method 1 is a simple count, Method 2 is an average count per parcel, Method 3 is an average count per unit area, and Method 4 is proportion of area with a variable present.

^a^N for pairwise comparisons involving public nuisances is 885 due to data availability.

^b^Crime is not constructed using Methods 2 or 4 as crime is measured at the block level.
