# Supplementary material for: Implications of construction method and spatial scale on measures of the built environment
Source: Int J Health Geogr. 2016 Apr 28;15:15. doi: 10.1186/s12942-016-0044-x (PMC4849096; doi:10.1186/s12942-016-0044-x)
Supplement: Supplementary file 2 — 10.1186/s12942-016-0044-x R code for constructing PACs and SACs from a shapefile. [file 12942_2016_44_MOESM2_ESM.docx]

**Additional File 2**

**Implications of Construction Method and Spatial Scale on Measures of the Built Environment**

Julie Strominger, Rebecca Anthopolos, and Marie Lynn Miranda

**R code for constructing PACs and SACs from a shapefile**

# Load rgdal and spdep packages

library(rgdal)

library(spdep)

# Read in shapefile

path_shp08<-‘S:/Projects/BE/Blocks/CAP_08.shp’

lyr08<-ogrListLayers(path_shp08)

shp08<-readOGR(path_shp08,layer=lyr08)

# Extract data from shapefile and plot

shp08_data<- shp08@data

plot(shp08)

# Extract out unique areal unit (GEOID10 in this example)

unq08<-data.frame(shp08$GEOID10)

names(unq08)<-c(“GEOID10”)

### Create PACs (i.e., neighbor list based on first-order adjacency)

pac08<-poly2nb(shp08,row.names= shp08$GEOID10)

# From list, create matrix flagging if certain block is in a given block’s PAC

pac08_mx<-nb2mat(pac08, style=”B”, zero.policy=T)

# Insert in 1s on the diagonal to ensure that each block contributes to own PAC

pac08_mx[row(pac08_mx)==col(pac08_mx)]<-1

# Compute number of blocks contributing to each PAC

nBlkpac<-apply(pac08_mx,2,sum)

# Create data frame from matrix

pac08_df<-data.frame(pac08_mx)

# Combine data frame with name of blocks, rename matrix columns to be corresponding block

pac08_df<-cbind(unq08, pac08_df)

names(pac08_df)<-c(“GEOID10”, paste(unq08$GEOID10))

### Create SACs (i.e., neighbor list based on second-order adjacency)

sac08_a<-nblag(pac08, 2)

# Combine lists for higher order adjacencies

sac08<-nblag_cumul(sac08_a)

# From combined list, create matrix flagging if certain block is in a given block’s SAC

sac08_mx<-nb2mat(sac08, style=”B”, zero.policy=T)

# Insert in 1s on the diagonal to ensure that each block contributes to own SAC

sac08_mx[row(sac08_mx)==col(sac08_mx)]<-1

# Compute number of blocks contributing to each SAC

nBlksac<-apply(sac08_mx,2,sum)

# Create data frame from matrix

sac08_df<-data.frame(sac08_mx)

# Combine data frame with name of blocks, rename matrix columns to be corresponding block

sac08_df<-cbind(unq08, sac08_df)

names(sac08_df)<-c(“GEOID10”, paste(unq08$GEOID10))
